# Antimicrobial Stewardship Program, COVID-19, and Infection Control: Spread of Carbapenem-Resistant Klebsiella Pneumoniae Colonization in ICU COVID-19 Patients. What Did Not Work?

**DOI:** 10.3390/jcm9092744

**Published:** 2020-08-25

**Authors:** Beatrice Tiri, Emanuela Sensi, Viola Marsiliani, Mizar Cantarini, Giulia Priante, Carlo Vernelli, Lucia Assunta Martella, Monya Costantini, Alessandro Mariottini, Paolo Andreani, Paolo Bruzzone, Fabio Suadoni, Marsilio Francucci, Roberto Cirocchi, Stefano Cappanera

**Affiliations:** 1 Antimicrobial Stewardship Unit, Department of medicine, St. Maria Hospital, 05100 Terni, Italy; tiri.beatrice@gmail.com; 2 Department of Critical Care Medicine and Anesthesiology, St. Maria Hospital, 05100 Terni, Italy; e.sensi@aospterni.it (E.S.); v.marsiliani@aospterni.it (V.M.); m.cantarini@aospterni.it (M.C.); 3 Infectious Diseases Clinic, Department of medicine, St. Maria Hospital, 05100 Terni, Italy; giulia.priante1989@gmail.com (G.P.); c.vernelli@aospterni.it (C.V.); l.martella@aospterni.it (L.A.M.); 4 Pharmacy Unit, St. Maria Hospital, 05100 Terni, Italy; m.costantini@aospterni.it; 5 Hematology and Microbiology Laboratory, St. Maria Hospital, 05100 Terni, Italy; a.mariottini@aospterni.it (A.M.); p.andreani@aospterni.it (P.A.); 6 Department of General and Specialist Surgery “Paride Stefanini”, 00185 Rome, Italy; paolo.bruzzone@uniroma1.it; 7 Section of Legal Medicine, St. Maria Hospital, 05100 Terni, Italy; f.suadoni@aospterni.it; 8 Department of General and Oncologic Surgery, St. Maria Hospital, 05100 Terni, Italy; m.francucci@aospterni.it; 9 Department of General and Oncologic Surgery, University of Perugia, St. Maria Hospital, 05100 Terni, Italy; roberto.cirocchi@unipg.it

**Keywords:** *carbapenem-resistant Klebsiella pneumoniae*, antimicrobial stewardship, *CRE* colonization, COVID-19, intensive care unit

## Abstract

The Italian burden of disease associated with infections due to antibiotic-resistant bacteria has been very high, largely attributed to *Carbapenem-Resistant Klebsiella pneumoniae* (*CR-Kp*). The implementation of infection control measures and antimicrobial stewardship programs (ASP) has been shown to reduce healthcare-related infections caused by multidrug resistance (MDR) germs. Since 2016, in our teaching hospital of Terni, an ASP has been implemented in an intensive care unit (ICU) setting, with the “daily-ICU round strategy” and particular attention to infection control measures. We performed active surveillance for search patients colonized by *Carbapenem-Resistant Enterobacteriaceae* (*CRE*). In March 2020, coronavirus disease 2019 (COVID-19) arrived and the same ICU was reserved only for COVID-19 patients. In our retrospective observational study, we analyzed the bimonthly incidence of *CRE* colonization patients and the incidence of *CRE* acquisition in our ICU during the period of January 2019 to June 2020. In consideration of the great attention and training of all staff on infection control measures in the COVID-19 era, we would have expected a clear reduction in *CRE* acquisition, but this did not happen. In fact, the incidence of *CRE* acquisition went from 6.7% in 2019 to 50% in March–April 2020. We noted that 67% of patients that had been changed in posture with prone position were colonized by *CRE*, while only 37% of patients that had not been changed in posture were colonized by *CRE*. In our opinion, the high intensity of care, the prone position requiring 4–5 healthcare workers (HCWs), equipped with personal protective equipment (PPE) in a high risk area, with extended and prolonged contact with the patient, and the presence of 32 new HCWs from other departments and without work experience in the ICU setting, contributed to the spread of *CR-Kp* in our ICU, determining an increase in *CRE* acquisition colonization.

## 1. Introduction

The European burden of disease associated with infections owing to antibiotic-resistant bacteria was very high as far as Italy was concerned, with 10,762 deaths in 2015 [1]. A large contribution was owing to *carbapenem-resistant Klebsiella pneumoniae* (*CR-Kp*), with a high mortality (30–70%) [2]. The implementation of infection control measures for reducing in-hospital transmission of *CR-Kp* and of antimicrobial stewardship programs have been shown to reduce healthcare-related infections caused by multidrug resistance (MDR) germs [3]. The European Centre for Disease Control (ECDC) reported that, in Europe, 7.5% of *Klebsiella pneumoniae* isolated from blood cultures and cerebral spinal fluid were resistant to carbapenems, while in Italy, it was 26.8% [4]. During the ECDC visit to Italy in order to discuss antimicrobial resistance (AMR) issues, it was reported that the AMR situation is one of the major Italian health problems, hence warning to urgently introduce measures for the appropriate use of the antibiotics and to improve infection control [5]. Organization for Economic Co-operation and Development (OECD) declared that, in Italy, there are higher than average rates of healthcare associated infections and the total volume of antibiotics prescribed is the second highest in the OECD. In our 554-bed teaching hospital, an antimicrobial stewardship program (ASP) has been active since 2016, with particular attention to the intensive care unit (ICU) setting and infection control measures. In March 2020, COVID-19 arrived and the infection control became essential to prevent the spread of Sars-CoV-2. In this article, we report an observational retrospective analysis to evaluate the incidence of *Carbapenem-Resistant Enterobacteriaceae* (*CRE*) colonization and *CRE* acquisition in the ICU in pre-COVID-19 era, during the COVID-19 pandemic, and in post-COVID-19 era.

## 2. Materials and Methods

### 2.1. Setting

Since 2016, in our teaching hospital of Terni (Umbria, Italy), with 554 hospital beds, an ASP has been implemented in our ICU setting with 14 beds. The ICU cares for a mixed population of post-surgical patients and patients suffering from life-threatening respiratory and cardiovascular conditions as well as trauma, sepsis shock, and so on. The core strategy of our ASP was a “daily-ICU round strategy”, in which the infectious disease expert in antimicrobial stewardship takes review of antibiotics daily, discussing them with the intensivist about de-escalation, withdraw when infection was not confirmed, stopping antibiotic therapy for the end of treatment, using a nudge approach.

### 2.2. Infection Control Policy

Particular attention was given to infection control practices with hand hygiene intervention, definition by checklist and continuous training on contact precautions for *CRE* carriers. We also performed active surveillance through systematic screening with rectal swab and clinical culture to search patients colonized by *CRE*, performed on all new ICU admissions and tested again on the seventh day of hospitalization. Bimonthly data were shared with ICU staff in regular meetings [3].

The surveillance test to detect *CRE* on rectal swab was a culture-based method with a direct inoculation into specific selective chromogenic media. Growing colonies were identified by Vitek-MS, matrix-assisted laser desorption/ionization time-of-flight (MALDI-TOF, bioMeriéux), and tested for carbapenems resistance and antibiotic susceptibility using the Vitek 2 system (bioMerieux, Marcy-L’Étoile, France). Enterobacteriaceae colonies were also analyzed by immunochromatography test, for the identification of OXA-48-like, OXA carbapenemase (OXA-163), Klebsiella Pneumoniae Carbapenemase (KPC), New Delhi Metallo-beta-lactamase (NDM), and Verona Imipenemase (VIM) carbapenemases (RESIST-5 O.O.K.N.V. CORIS BioConcept, Allschwil, The Switzerlands).

### 2.3. COVID-19 Situation in Italy

In March 2020, Sars-CoV-2 arrived in our hospital. The same ICU became an ICU only for COVID-19 patients. All the healthcare workers (HCWs) in COVID-19 ICU wore personal protective equipment (PPE) in accordance with the World Health Organization (WHO) and Italian guidelines [6,7].

During the pandemic period, the number of HCWs in the ICU increased, in 24 h of work, 33 HCWs changed shifts. Therefore, 32 new members, from other departments and without work experience in the ICU setting, started working in the ICU.

All the patients admitted to the ICU received systematic screening for colonization of *CRE* through rectal swabbing or clinical culture (bronchoalveolar lavage or urine colture). During the ICU stay, the patients continued to be systematically screened through rectal swab for *CRE* colonization every 7 days and routine microbiological culture of bronchoalveolar lavage and urine culture. The patients with a site of colonization at the admission in the ICU were defined as primary colonization cases. The patients with “*CRE* acquisition” were considered the patients with a site of colonization obtained after a negative swab or clinical culture at ICU admission [8].

We analyzed the bimonthly incidence of *CRE* colonization patient and the incidence of *CRE* acquisition in our ICU from 1 January 2019 to June 2020. We analyzed the trend of the phenomenon before COVID-19 era, during the pandemic (March–April 2020) when the same ICU was reserved only for COVID-19 patients, and after COVID-19 era (May–June 2020) when the ICU was COVID-19 free.

## 3. Results

**Figure 1:**
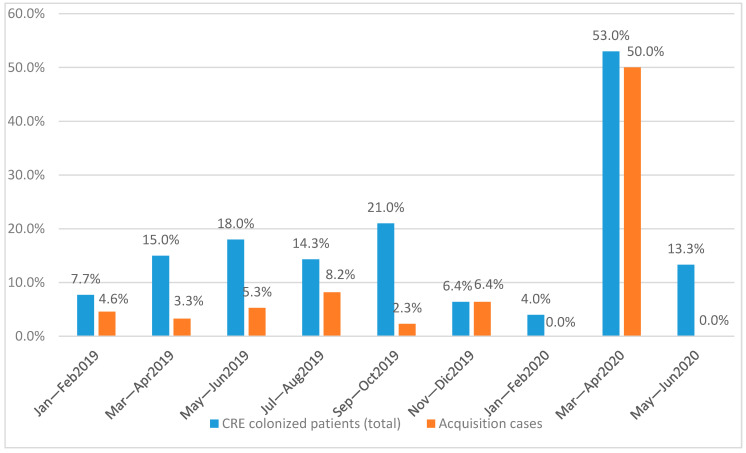
The trend of incidence of primary *Carbapenem-Resistant Enterobacteriaceae* (*CRE*) and acquisition *CRE* in intensive care unit (ICU).

We analyzed bimonthly data from January–February 2019 to May–June 2020 concerning the number of patients admitted to the ICU and number of rectal carriers of *CRE* (Table 1). We analyzed the trend of incidence of primary and *CRE* acquisition cases (Figure 1).

In the first two months (January and February) of 2019, 3.1% of cases were primary colonization cases and 4.6% *CRE* acquisition. Successively, in March–April 2019, the overall incidence was higher (15%), but the incidence rate of *CRE* acquisition cases was lower (3.3%). In May–June 2019, 5.3% of cases were *CRE* acquisition cases. In July–August 2019, *CRE* acquisition shown a slight increase in the incidence rate compared with the previous two months (8.2%). In September–October 2019, there was the highest incidence rate of *CRE* colonized patients, 21%, but the *CRE* acquisition was only 1, with the lowest incidence rate of 2.3%. In November–December 2019, 31 patients were admitted with 4 colonized *CRE* patients, of which 2 *CRE* were acquisition cases.

In January–February 2020, 25 patients were admitted with only 1 primary colonization case.

From January 2019 to February 2020, the patients admitted to the ICU were a mixed population of post-surgical patients (neurosurgery, abdominal surgery, urological surgery, thoracic surgery, and so on) or patients suffering from life-threatening conditions such as septic shock, acute respiratory failure, trauma, and so on. Patients may be admitted from surgical wards or the emergency department or from any ward if they had conditions that warranted intensive care. The ASP did “daily-ICU round strategy”, with the daily discussion about antibiotic therapy microbiological samples, and diagnosis of healthcare associated infections (HAIs) and infection control measures application.

On 20 February 2020 Sars-CoV-2 arrived in Italy and, on 4 March 2020, the first COVID-19 case arrived in our hospital.

In March 2020, the same ICU with 14 beds become an ICU only for COVID-19 patients. All the patients admitted had a Sars-CoV-2 isolation from a nasopharyngeal or oropharyngeal or bronchoalveolar sample and all patients needed ventilator support by oro-tracheal intubation. The HCWs in the COVID-19 ICU were trained on donning and doffing PPE. In accordance with the indications of the Ministry of Health, all staff were wearing gloves, eye protection, full coveralls, N95 respirators, or respirators that offer a higher level of protection and are worn for a session of work in a high risk area such as a COVID-19 ICU. The COVID-19 patients in ICU needed a high intensity of care, such as being changed in posture with the prone position for 12 to 16 h per day. About 4–5 HCWs were needed for posture changes of a patient. In 24 h of work in the COVID-19 ICU, 33 HCWs changed shifts, of which 32 were from other departments and without work experience in the ICU setting.

In March–April 2020, 32 patients were admitted to COVID-19 ICU for Sars-CoV-2-related acute respiratory distress syndrome (ARDS). Of these, 17 patients were *CRE* colonization: 1 was defined as a primary colonization case and 16 were defined as *CRE* acquisition with an incidence of 50%. Analyzing the trend of incidence of primary and *CRE* acquisition, we can say that, before the COVID-19 era, the average of incidence of primary cases was 12.3% (range 4–14.3%) and the average *CRE* acquisition was 4.3% (range 0–8.2%) with a constant trend. In the COVID-19 ICU (March–April 2020), the incidence of *CRE* acquisition increased significantly, as shown in Figure 1.

In May–June 2020, the ICU was COVID-19 free. During this period, 30 patients were admitted, of which 4 were colonized patients, all defined as primary cases. The analysis of the incidence of the acquired cases showed a decreasing trend from 50% to 0%.

**Figure 2:**
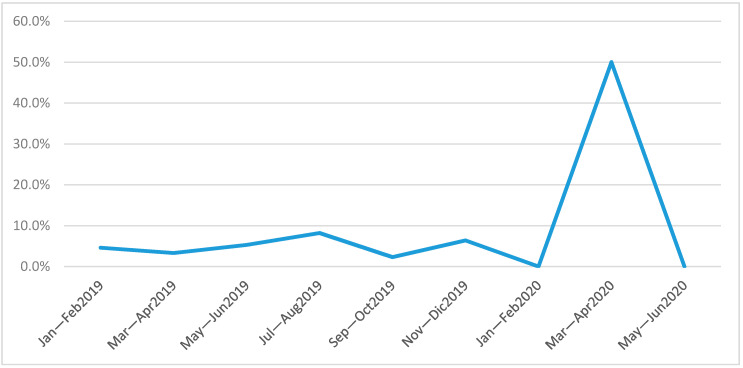
The trend of incidence of *CRE* acquisition colonization.

The trend of incidence of *CRE* acquisition colonization is shown in Figure 2.

**Figure 3:**
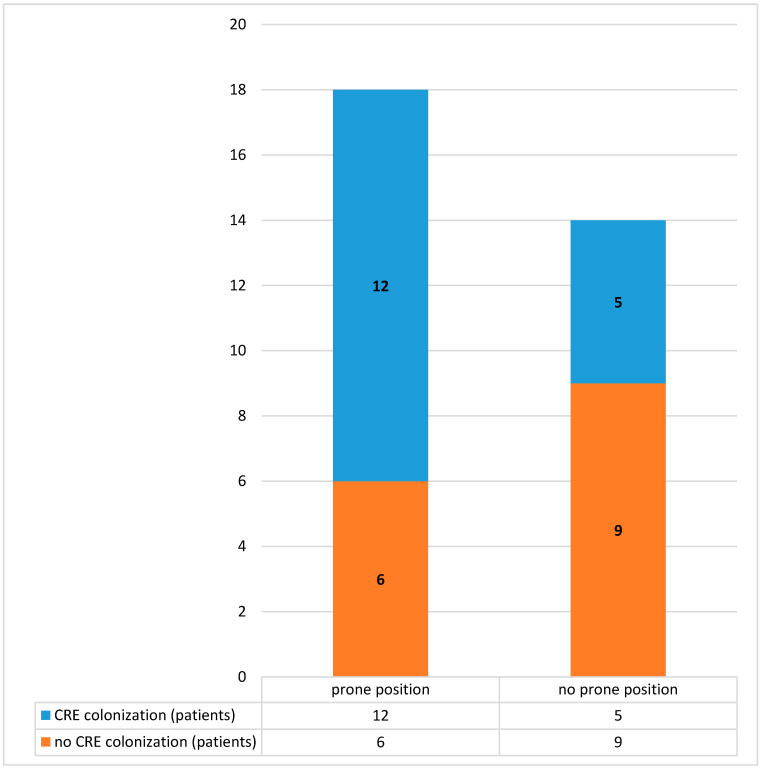
Data about the acute respiratory distress syndrome (ARDS) coronavirus disease 2019 (COVID-19) patients pronate and not pronate and *CRE* colonization.

All the *CRE* isolated were *Klebsiella pneumoniae KPC* (*Klebsiella Pneumoniae Carbapenemase*). Out of the 32 ARDS COVID-19 patients, 18 were pronate and 14 were not. Of the 18 pronate patients, about 12 were colonized by *CRE* (1 primary colonization cases and 11 *CRE* acquisition cases). Out of the 14 not pronate patients, 5 were colonized by *CRE* and all cases were *CRE* acquisition cases. The data are reported in Figure 3.

## 4. Discussion

Curbing the spread of *CRE* into healthcare facilities is important as is controlling transmission in areas where they have become endemic, such as Italy, because they are associated with poor patient outcomes. Implementing infection control measures is an important step in order to prevent patients from becoming colonized or infected by *CRE*.

The European Society of Clinical Microbiology and Infectious Diseases (ESCMID) guidelines for the management of infection control measures to reduce transmission of multidrug-resistant Gram-negative bacteria in hospital patients define the implementation of hand hygiene education programmes, contact precautions, use of alert code to identify promptly patients colonized by *CRE*, isolate colonized and infected patients, implementation of a programme of active screening culture, and implementation of an antimicrobial stewardship program as a strong recommendation [3]. All these recommendations have been implemented in our ASP active in the ICU since 2016. In 2019, the average incidence rate of *CRE* acquisition cases in our ICU was 5%.

In a few months, COVID-19 has transformed the world. The situation in Italy has been made dramatic in all social contexts [9]. Infection control becomes essential to contain and mitigate the risk of nosocomial transmission and outbreak; therefore, all suspected cases in our hospital were isolated for contact, droplet, and airborne precautions.

In March 2020, the intensive care unit became a department dedicated exclusively to COVID-19 intubated patients, defined as a high risk setting for the risk of transmission to healthcare workers. All the HCWs were trained on donning and doffing PPE, all staff had access to the PPE to protect themselves, gloves and aprons were subject to single use as with disposal after each patient or resident contact, fluid repellent surgical mask and eye protection were used for a session of work, gowns or coveralls were worn for a session of work in higher risk areas, and hand hygiene was practiced and extended to exposed forearms after removing any element of PPE [10]. Considering the high number of HCWs employed (in 24 h of work in the COVID-19 ICU, 33 HCWs worked in shifts), we can affirm that the use of PPE was effective in order to protect them because no healthcare professionals tested were positive for Sars-CoV-2.

In consideration of the great attention and training of all staff on infection control measures, we would have expected a clear reduction in *CRE* acquisition, but this did not happen; in fact, the incidence of *CRE* acquisition went from 5% on average in 2019 to 50% during the pandemic (March–April 2020). Therefore, the use of PPE was correct and protected the HCWs, but this did not protect patients from *CRE* acquisition colonization. During the pandemic period, HCWs from other departments without work experience in the ICU setting started working in ICU.

In addition, we noted that the 67% of patients that were changed in posture with prone position were colonized by *CRE*, while the 37% of patients that were not changed in posture were colonized by *CRE*. The mechanism of *CRE* acquisition is the microbe cross-transmission, which is summarized by the World Health Organization (WHO) with the presence of microbes in patient, transfer of this organism to HCWs, and cross-transmission to other patients [11].

Incorrect hand cleaning during patient care has been demonstrated to facilitate the cross-transmission of Gram negative bacteria (GNB) [12]. GNB survive on hands for periods lasting from a few minutes to several hours [13]. Hand contamination despite wearing gloves has been reported in 4.5% and 1% of HCWs [14]. It has also been demonstrated that the *Klebsiella* spp. contamination of staff took place after simple procedures and the GNB counts on HCWs are related to the type of contact [15,16].

The clothing of healthcare staff can be a source for cross-transmission of healthcare-associated pathogens [17,18,19,20,21,22]. The contamination of gowns and gloves has been shown to be a frequent event during patient care; transfer of *Klebsiella* spp. to staffs’ hands took place after simple “clean” procedures such as washing the patients and touching several parts of the body during daily activities [14,23,24].

The COVID-19 patients recovering in the ICU are patients with a high intensity of care, with a continuous need for assistance, and thus a continuous need to be touched by the HCWs, such as the need to be pronated. To change a patient in prone position required the employment of 4–5 HCWs equipped with all the necessary PPE. The incidence rate of colonized *CRE* patients was higher in patients pronated (67%) rather than in not pronated. The HCWs, in accordance with the indication of the Ministry of Health, must wear gowns or coveralls for a session of work because they are in higher risk areas.

Therefore, in our opinion, the high intensity of care, the need to be changed in prone position with the employment of 4–5 HCWs equipped with PPE in a high risk area with extended and prolonged contact with the patient, and the presence of health personnel without work experience in ICU setting regarding contact precautions contributed to the spread of *Klebsiella pneumoniae KPC* in our ICU, determining an increased in *CRE* acquisition colonization.

After the pandemic, the type of patients and ICU staff members returned to that of the pre COVID-19 era. The result was that the incidence of *CRE* acquisition went from 50% to 0%.

## 5. Conclusions

Working during a pandemic event in a high risk setting like the COVID-19 ICU is more difficult because of the use of PPE. The ARDS Sars-CoV-2 patients are patients requiring high intensity of care with the employment of many HCWs. Many COVID-19 patients needed to be changed to prone position for 12 to 16 h per day, and this maneuver creates prolonged and extended contact between the patient and the operator. Our study highlighted how this is probably the root cause of the *Klebsiella pneumoniae KPC* spread in the COVID-19 ICU.

## Figures and Tables

**Table 1:** Bimonthly data from January 2019 to June 2020 about number of patients admitted to intensive care unit (ICU) and number of *Carbapenem-Resistant Enterobacteriaceae* (*CRE*) colonization.

	Patients (Total)	CRE Colonized Patients (N)	CRE Colonized Patients (%)	Primary Cases (Patients)	Acquisition Cases (Patients)	% Acquisition Cases (Patients)
Jan—Feb 2019	65	5	7.7%	2	3	4.6%
Mar—Apr 2019	60	9	15.0%	7	2	3.3%
May—Jun 2019	56	10	18.0%	7	3	5.3%
Jul—Aug 2019	49	7	14.3%	3	4	8.2%
Sep—Oct 2019	43	9	21.0%	8	1	2.3%
Nov—Dec 2019	31	2	6.4%	2	2	6.4%
Jan—Feb 2020	25	1	4.0%	1	0	0.0%
Mar—Apr 2020	32	17	53.0%	1	16	50.0%
May—Jun 2020	30	4	13.3%	4	0	0.0%
